# Household air pollution from use of cooking fuel and under-five mortality: The role of breastfeeding status and kitchen location in Pakistan

**DOI:** 10.1371/journal.pone.0173256

**Published:** 2017-03-09

**Authors:** Sabrina Naz, Andrew Page, Kingsley Emwinyore Agho

**Affiliations:** 1 Centre of Health Research, School of Medicine, Western Sydney University, Campbelltown Campus, Penrith, New South Wales, Australia; 2 School of Science and Health, Western Sydney University, Campbelltown Campus, Penrith, New South Wales, Australia; Liverpool School of Tropical Medicine, UNITED KINGDOM

## Abstract

Household air pollution (HAP) mainly from cooking fuel is one of the major causes of respiratory illness and deaths among young children in low and middle-income countries like Pakistan. This study investigates for the first time the association between HAP from cooking fuel and under-five mortality using the 2013 Pakistan Demographic and Health Survey (PDHS) data. Multi-level logistic regression models were used to examine the association between HAP and under-five mortality in a total of 11,507 living children across four age-groups (neonatal aged 0–28 days, post-neonatal aged 1–11 months, child aged 12–59 months and under-five aged 0–59 months). Use of cooking fuel was weakly associated with total under-five mortality (OR = 1.22, 95%CI = 0.92–1.64, *P* = 0.170), with stronger associations evident for sub-group analyses of children aged 12–59 months (OR = 1.98, 95%CI = 0.75–5.25, *P* = 0.169). Strong associations between use of cooking fuel and mortality were evident (ORs >5) in those aged 12–59 months for households without a separate kitchen using polluting fuels, and in children whose mother never breastfed. The results of this study suggest that HAP from cooking fuel is associated with a modest increase in the risk of death among children under five years of age in Pakistan, but particularly in those aged 12–59 months, and those living in poorer socioeconomic conditions. To reduce exposure to cooking fuel which is a preventable determinant of under-five mortality in Pakistan, the challenge remains to promote behavioural interventions such as breastfeeding in infancy period, keeping young children away from the cooking area, and improvements in housing and kitchen design.

## Introduction

Pakistan is the sixth most populous country in the world located in north-west South Asia [1]. Pakistan is an agricultural country, and nearly 64% of the population mainly live in rural areas [1], where access to commercial and clean energy resources is limited and traditional ways of using solid fuels (such as wood, straw/shrubs/grass, animal dung, charcoal, and coal) are the only available options for domestic cooking fuel [2]. Almost 87% of rural and 13% of urban households in Pakistan use solid fuels for cooking [1], and when these fuels are burnt in open fire produces health-damaging pollutants and chemicals. [3–5].

Household air pollution (HAP) from unprocessed fuel is a substantial cause of respiratory illness and death and remains a major public health concern in low and middle-income countries [6], and globally almost 4.3 million deaths annually have been attributed to HAP [7]. Children less than five years of age are the most vulnerable to HAP related illness such as respiratory infections, due to their proximity to domestic cooking [8–10]. Pneumonia, which is caused by ARI, is still a leading cause of death among children under age five in Pakistan [1]. The under-five mortality rate in Pakistan declined from 139 per 1,000 live births in 1990 to 86 in 2013 [11]; which remains above the Millennium Development Goal 4 (MDG4) target that is to reduce under-five mortality by two-thirds (46 per 1,000 live births) [12]. Disability-adjusted life years (DALYs) from the Global Burden of Diseases (GBD) study shows that 10% of the total burden of disease for all ages in the country has been associated with ARI [13], and according to the World Health Organization (WHO) 9% of the total burden of diseases in Pakistan has been attributable to HAP [14].

There have been few studies from Pakistan investigating the association between HAP and the health of young children, with most limited to a particular health outcome (e.g. low birth weight/acute respiratory infection) or specific geographical area or regional population. [3, 15–17], Only two studies have examined the effect of HAP on non-fatal respiratory diseases among under-five children [3, 17]. To date, no previous studies from Pakistan have examined the effect of HAP from use of cooking fuel on under-five mortality using large-scale and nationally representative data, and also have not considered the role of potential environmental and behavioural factors that might affect the level of exposure to cooking fuel. Accordingly, this study investigated the association between HAP from the use of cooking fuel and under-five mortality (classified as neonatal 0–28 days, post-neonatal 1–11 months, child 12–59 months and under-five 0–59 months), and assessed how observed associations were affected by key environmental and behavioural factors in Pakistan.

## Materials and methods

### Data sources

The most recent nationally representative Pakistan Demographic and Health Survey (PDHS) dataset for the year 2013 was used for this study, collected with approval from the DHS program (http://www.dhsprogram.com/). The PDHS was conducted in Pakistan with the collaboration of the global DHS program. The survey was implemented by the National Institute of Population Studies (NIPS) with technical and logistical support from the ICF international of Calverton, Maryland, USA and financial support provided by the United States Agency for International Development (USAID) [1]. Demographic and heath data of urban and rural areas were collected by interviewing ever-married women and men aged 15–49 years using a stratified sample of households based on a two-stage cluster design [1].

A total of 13,558 eligible women of reproductive age of 15–49 years were included in the sample with a response rate of 93.1%. This present study was based on information related to 11,507 singleton live-born children, of whom 768 died in the 5-years prior to the survey date. Data relating to under-five mortality (classified as neonatal 0–28 days, post-neonatal 1–11 months, child 12–59 months and under-five 0–59 months) was restricted to this period of five years to minimise recall bias of child birth and death information which was self-reported by the mother.

### Study outcomes

The analysis for under-five mortality was carried out for three separate age groups (as a proportion of total live births): neonatal mortality (number of deaths during the first 28 days of life, 0–28 days), post-neonatal mortality (number of deaths between one month and the first birthday, 1–11 months), child mortality (number of deaths between age one and five, 12–59 months). Analyses combining these three groups for under-five mortality (number of deaths between birth and the fifth birthday, 0–59 months) were also conducted.

The outcome variables were dichotomous for the analysis, where age at death was either ‘yes’ (= 1) or ‘no’ (= 0).

### Exposure to cooking fuel

The type of cooking fuel was the main exposure variable in this study. In PDHS, the mothers were asked, “What type of fuel does your household mainly use for cooking?”, and 10 types of cooking fuels were reported in response. These fuels were grouped into two categories in this analysis on the basis of exposure to cooking smoke: “clean fuels” including electricity, liquid petroleum gas (LPG), natural gas and biogas and “polluting fuels” including kerosene, coal/lignite, charcoal, wood, straw/shrubs/grass and animal dung. Kerosene was categorised in the polluting fuels group in this study as some previous studies on HAP have reported kerosene as a polluting fuel and have shown significant associations between under-five mortality or respiratory illness among young children and use of kerosene fuel in the household [18–21].

### Potential confounders

A number of indicators of socio-economic status were incorporated in the study including, place of residence (categorized as “urban” or “rural”), household wealth index (“high income”, “middle income” or “low income”), mother’s education (“secondary or higher”, “primary” or “no education”), mother’s working status (“not working” or “working”), smoking status of mother (categorized as “yes” or “no”), floor material of household (“cement/carpet” or “earth/sand”) and wall material of household (“cement/brick” or “non-cement/non-brick”). These socio-economic status variables have previously been described as potential confounders for the association between HAP and under-five mortality [20–30]. “Low income” represented the bottom 40% of households, “middle income” represented the middle 40% of households, and “high income” represented the top 20% of households, based on the method defined by Filmer and Pritchett [31]. Mother’s age (categorised as <30, 30–39 and 40–49 years) and sex of the child (“female” or “male”) were also considered as covariates in this study.

Breastfeeding has previously been presented to provide important protection against infectious diseases and may be associated with lower under-five mortality, especially in neonatal and infancy period [21, 23, 25, 26, 32–36] and reduce the risk of disease associated with HAP. Likewise, a household without a separate kitchen for cooking has also previously been associated as a proxy measure of a greater level of exposure to use of cooking fuel, based on proximity to polluting fuels [25, 28, 37–41]. Breastfeeding status of children (categorised as ever breastfed “yes” or “no”) and location of the kitchen either inside the house or separate from buildings/outdoors (categorised as separate kitchen “yes” or “no”) were taken into account *a priori* as factors that may indicate different levels of exposure to polluting fuels. (Descriptive characteristics of study factors are provided in a S1 Table).

### Statistical analysis

Neonatal, post-neonatal, child and under-five mortality incidence proportions were calculated by following a similar approach of the DHS program provided by Rutstien and Rojas [42]. Multilevel logistic regression models were used to investigate the association between type of cooking fuels and neonatal, post-neonatal, child and under-five mortality adjusted for the selected covariates of place of residence, wealth index, mother’s age, mother’s education, mother’s working status, sex of child, breastfeeding status, wall material and floor material of household, location of kitchen and smoking status of mothers.

Analyses stratified by breastfeeding status and by the location of the kitchen were also conducted to define whether the magnitude of the effect of the cooking fuel on mortality outcomes differed across different levels of these two variables. Breastfeeding status and location of the kitchen were each combined with the type of cooking fuel as composite ordinal variables to examine different levels of exposure to cooking fuel for neonatal, post-neonatal, child and under-five mortality outcomes.

The weighted incidence proportion estimates of mortality were calculated using the “SVY” command to adjust for the cluster sampling survey design and weights. Random effects multilevel logistic regression models were conducted by using the “xtlogit” command. All analyses were carried out in STATA version 13.1 (Stata Corp: College Station, TX, USA).

### Ethics

Before the Demography and Health Survey (DHS) were conducted, this survey sought and obtained the required ethical approvals from ethics committees in Pakistan. Informed consent was obtained from study participants before their participation in the surveys. Publicly available, de-identified datasets were used in this study following approval from The DHS Program to download and use the data.

## Results

**Fig 1:**
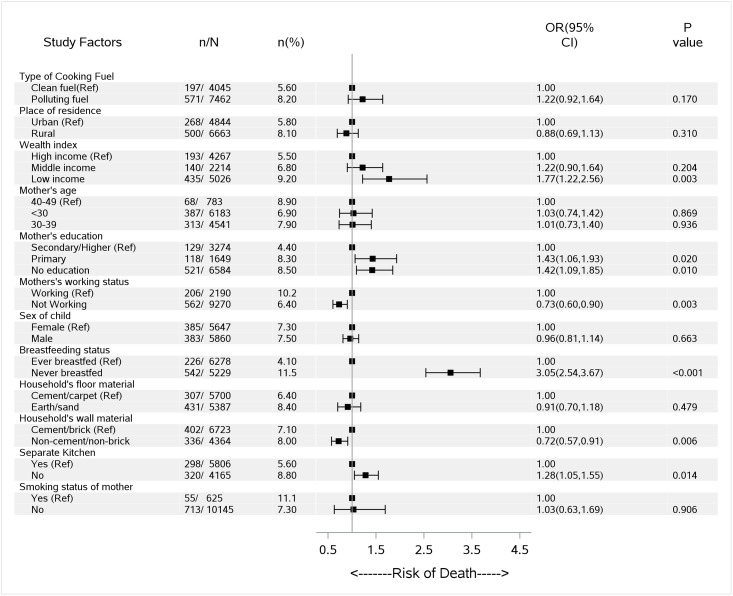
Use of cooking fuel associated with total under-five mortality in Pakistan. Ref = Reference category; n = number of under-five mortality cases and N = total number of under-five children; n(%) = weighted incidence proportion of under-five mortality cases; OR (95% CI) = odds ratio adjusted for wealth index, place of residence, mother’s age, education and working status, sex of child, breastfeeding status, household’s floor material and wall material, location of kitchen and smoking status of mother; clean fuels = electricity, liquid petroleum gas (LPG), natural gas, biogas; Polluting fuels = kerosene, coal/lignite, charcoal, wood, straw/shrubs/grass and animal dung.

Use of polluting fuels (wood, straw/shrubs/grass, animal dung, charcoal, coal/lignite, kerosene) for cooking was associated with a higher risk of child mortality (OR = 1.98, 95%CI = 0.75–5.25, *P* = 0.169), after adjusting for place of residence, household wealth, mother’s age, mother’s education, mother’s working status, sex of child, breastfeeding status, floor and wall material of household, location of kitchen and smoking status of mother (Table 1). Effect sizes of smaller magnitude were evident for post-neonatal mortality (OR = 1.31, 95%CI = 0.75–2.27, *P* = 0.342) followed by neonatal mortality (OR = 1.09, 95%CI = 0.77–1.54, *P* = 0.643) (Table 1). The association between use of polluting fuels for cooking (compared to no polluting fuels) and overall under-five mortality was (OR = 1.22, 95%CI = 0.92–1.64, *P* = 0.170) after adjusting for covariates (Fig 1). A sub-analysis was conducted to investigate the association between different fuel type with under-five mortality and found slightly higher association for use of straw/shrubs/grass, animal dung (OR = 1.39, 95%CI = 0.96–2.01, *P* = 0.081) followed by use of kerosene, coal/lignite, charcoal (OR = 1.34, 95%CI = 0.72–2.49, *P* = 0.350) and use of wood (OR = 1.20, 95%CI = 0.89–1.60, *P* = 0.234) compared to use of electricity, LPG/natural gas, biogas associated with total under-five mortality in this study after adjustment for selected covariates (data not shown).

**Table 1:** HAP from cooking fuels associated with neonatal, post-neonatal and child mortality in Pakistan.

Study factors	Neonatal				Post-neonatal				Child			
	n ^a^	Deaths /1000 live births	OR ^b^ 95%CI	*P* value	n ^a^	Deaths /1000 live births	OR ^b^ 95%CI	*P* value	n ^a^	Deaths /1000 live births	OR ^b^ 95%CI	*P* value
**Type of Cooking Fuel**												
Clean fuel ^c^ ^d^	136	34.8	1.00		49	12.3	1.00		12	3.0	1.00	
Polluting fuel ^e^	367	51.7	1.09 (0.77–1.54)	0.643	145	19.8	1.31 (0.75–2.27)	0.342	59	8.0	1.98 (0.75–5.25)	0.169
**Place of Residence**												
Urban ^c^	178	38.2	1.00		63	13.2	1.00		27	5.6	1.00	
Rural	325	51.3	1.04 (0.77–1.40)	0.789	131	20.1	0.70 (0.45–1.09)	0.110	44	6.6	0.58 (0.29–1.14)	0.111
**Wealth Index**												
High income ^c^	136	32.9	1.00		44	10.4	1.00		13	3.1	1.00	
Middle income	95	44.8	1.20 (0.84–1.72)	0.324	35	16.1	1.31 (0.72–2.38)	0.379	10	4.5	0.80 (0.27–2.32)	0.677
Low income	272	57.2	1.58 (1.01–2.47)	0.047	115	23.4	1.89 (0.92–3.86)	0.083	48	9.6	2.07 (0.68–6.33)	0.201
**Mother's Age**												
40–49 ^c^	51	69.7	1.00		12	15.6	1.00		5	6.4	1.00	
<30	244	41.1	0.82 (0.57–1.20)	0.309	104	17.1	1.50 (0.73–3.06)	0.266	39	6.3	2.17 (0.65–7.20)	0.206
30–39	208	48.0	0.88 (0.60–1.27)	0.483	78	17.5	1.40 (0.69–2.86)	0.355	27	6.0	1.51 (0.44–5.14)	0.511
**Mother's Education**												
Secondary/ Higher ^c^	98	30.9	1.00		27	8.3	1.00		4	1.2	1.00	
Primary	77	49.0	1.19 (0.83–1.70)	0.351	33	20.4	1.88 (1.07–3.31)	0.029	8	4.9	3.45 (0.84–14.13)	0.085
No education	328	52.4	1.27 (0.92–1.74)	0.140	134	20.8	1.31 (0.77–2.22)	0.316	59	9.0	6.20 (1.77–21.80)	0.004
**Mother's Working Status**												
Working ^c^	135	65.7	1.00		50	23.4	1.00		21	9.7	1.00	
Not working	368	41.3	0.77 (0.60–0.99)	0.041	144	15.8	0.66 (0.45–0.97)	0.033	50	5.4	0.83 (0.44–1.57)	0.570
**Sex of Child**												
Female ^c^	255	47.3	1.00		97	17.5	1.00		33	5.9	1.00	
Male	248	44.2	0.90 (0.73–1.10)	0.308	97	16.8	1.10 (0.79–1.54)	0.563	38	6.5	1.09 (0.63–1.87)	0.760
**Breastfeeding Status**												
Ever breastfed ^c^	137	22.3	1.00		60	9.6	1.00		29	4.6	1.00	
Never breastfed	366	75.3	3.20 (2.55–4.02)	<0.001	134	26.3	2.54 (1.78–3.62)	<0.001	42	8.1	2.31 (1.32–4.05)	0.003
**Household’s Floor Material**												
Cement/carpet ^c^	211	38.4	1.00		67	11.9	1.00		29	5.1	1.00	
Earth/sand	273	53.4	0.89(0.65–1.22)	0.478	119	22.6	0.68 (0.44–1.04)	0.074	39	7.3	0.46 (0.22–0.95)	0.037
**Household’s Wall Material**												
Cement/brick ^c^	274	42.5	1.00		93	14.0	1.00		35	5.2	1.00	
Non-cement/non-brick	210	50.6	0.77 (0.58–1.01)	0.065	93	21.8	1.31 (0.79–2.16)	0.298	33	7.6	0.90 (0.46–1.77)	0.767
**Separate Kitchen**												
Yes ^c^	201	35.9	1.00		79	13.8	1.00		18	3.1	1.00	
No	210	53.1	1.29 (1.01–1.63)	0.037	72	17.6	1.01 (0.70–1.46)	0.973	38	9.2	2.12 (1.13–3.97)	0.019
**Smoking status of mother**												
Yes ^c^	28	46.9	1.00		25	41.7	1.00		2	3.2	1.00	
No	475	45.8	1.30 (0.68–2.48)	0.429	169	15.8	0.58 (0.27–1.24)	0.162	69	6.4	2.00 (0.27–14.76)	0.496

^a^ n = number of mortality cases for neonatal, post-neonatal and child age-groups;

^b^ odds ratio adjusted for wealth index, place of residence, mother’s age, mother’s education, mother’s working status, sex of child, breastfeeding status, household’s floor material, household’s wall material, separate kitchen in the house and smoking status of mother;

^c^ reference category,

^d^ clean fuels: electricity, liquid petroleum gas (LPG), natural gas, biogas,

^e^ Polluting fuels: kerosene, coal/lignite, charcoal, wood, straw/shrubs/grass and animal dung.

Stratified analyses to investigate different levels of exposure to use of cooking fuel found more than 3-fold higher risk of under-five mortality in children whose mother never breastfed and used polluting fuels for cooking (compared to breastfeeding mother who used clean fuels), with similar associations evident for neonatal mortality (OR = 3.34, 95%CI = 2.21–5.04, *P*<0.001) and post-neonatal mortality (OR = 3.39, 95%CI = 1.81–6.36, *P*<0.001) (Tables 2 & 3). Similarly, analyses combining location of kitchen (separate room used as kitchen or not) and use of cooking fuel showed strong associations for households who used polluting fuels and had no separate kitchen (compared to households with a separate kitchen who used clean fuel for cooking), for child mortality (OR = 7.63, 95%CI = 2.08–27.95, *P* = 0.002), and associations of a smaller magnitude for under-five mortality (OR = 1.88, 95%CI = 1.40–2.53, *P*<0.001), post-neonatal mortality (OR = 1.78, 95%CI = 1.01–3.14, *P* = 0.045) and neonatal mortality (OR = 1.62, 95%CI = 1.14–2.31, *P* = 0.007) (Tables 2 & 3).

**Table 2:** Risk of neonatal and post-neonatal mortality by breastfeeding status and kitchen location.

Study Factors	Neonatal				Post-neonatal			
	n ^a^	Deaths/1000 live births	OR ^b^ 95%(CI)	*P* value	n ^a^	Deaths/1000 live births	OR ^b^ 95%(CI)	*P* value
**Combined Association of Breastfeeding Status and Use of Cooking fuel** ^f^								
Ever breastfed & used clean fuels ^c^ ^d^	40	20.3	1.00		16	8.0	1.00	
Ever breastfed & used polluting fuels ^e^	97	23.2	0.94 (0.60–1.46)	0.771	44	10.4	1.19 (0.61–2.33)	0.608
Never breastfed & used clean fuels ^d^	96	49.4	2.55 (1.70–3.81)	<0.001	33	16.5	1.76 (0.91–3.39)	0.092
Never breastfed & used polluting fuels ^e^	270	92.4	3.34 (2.21–5.04)	<0.001	101	32.7	3.39 (1.81–6.36)	<0.001
**Combined Association of Kitchen Location and Use of Cooking fuel** ^g^								
Separate kitchen used clean fuels ^c^ ^d^	91	33.2	1.00		29	10.3	1.00	
Separate kitchen used polluting fuels ^e^	110	38.4	1.10 (0.78–1.55)	0.598	50	17.1	1.58 (0.92–2.72)	0.100
No separate kitchen used clean fuels ^d^	31	40.4	1.19 (0.77–1.84)	0.439	10	12.7	1.03 (0.49–2.18)	0.940
No separate kitchen used polluting fuels ^e^	179	56.2	1.62 (1.14–2.31)	0.007	62	18.8	1.78 (1.01–3.14)	0.045

^a^ n = number of mortality cases for neonatal and post-neonatal age-groups;

^b^ adjusted odds ratio;

^c^ reference category;

^d^ clean fuels: electricity, liquid petroleum gas (LPG), natural gas, biogas;

^e^ Polluting fuels: kerosene, coal/lignite, charcoal, wood, straw/shrubs/grass and animal dung;

^f^ analyses adjusted for mother’s age, mother’s education, place of residence and location of kitchen and

^g^ analyses adjusted for mother’s age, mother’s education, place of residence and breastfeeding status.

**Table 3:** Risk of child and under-five mortality by breastfeeding status and kitchen location.

Study Factors	Child				Under-five			
	n ^a^	Deaths/1000 live births	OR ^b^ 95%(CI)	*P* value	n ^a^	Deaths/1000 live births	OR ^b^ 95%(CI)	*P* value
**Combined Association of Breastfeeding Status and Use of Cooking fuel** ^f^								
Ever breastfed & used clean fuels ^c^ ^d^	4	2.0	1.00		60	30.8	1.00	
Ever breastfed & used polluting fuels ^e^	25	5.9	2.42 (0.67–8.80)	0.179	166	40.4	1.09 (0.76–1.57)	0.631
Never breastfed & used clean fuels ^d^	8	3.9	2.41 (0.60–9.72)	0.217	137	72.1	2.38 (1.70–3.33)	<0.001
Never breastfed & used polluting fuels ^e^	34	10.8	5.11 (1.44–18.17)	0.012	405	145.4	3.65 (2.60–5.12)	<0.001
**Combined Association of Kitchen Location and Use of Cooking fuel** ^g^								
Separate kitchen used clean fuels ^c^ ^d^	3	1.1	1.00		123	45.4	1.00	
Separate kitchen used polluting fuels ^e^	15	5.1	4.11 (1.11–15.23)	0.035	175	62.6	1.32 (0.99–1.76)	0.061
No separate kitchen used clean fuels ^d^	6	7.6	5.28 (1.28–21.69)	0.021	47	62.5	1.27 (0.8681.83)	0.195
No separate kitchen used polluting fuels ^e^	32	9.6	7.63 (2.08–27.95)	0.002	273	88.3	1.88 (1.40–2.53)	<0.001

^a^ n = number of mortality cases for child and under-five age-groups;

^b^ adjusted odds ratio;

^c^ reference category;

^d^ clean fuels: electricity, liquid petroleum gas (LPG), natural gas, biogas;

^e^ Polluting fuels: kerosene, coal/lignite, charcoal, wood, straw/shrubs/grass and animal dung;

^f^ analyses adjusted for mother’s age, mother’s education, place of residence and location of kitchen and

^g^ analyses adjusted for mother’s age, mother’s education, place of residence and breastfeeding status.

## Discussion

Findings from this study suggested that HAP from cooking fuel was associated with neonatal, post-neonatal, child and under-five mortality after adjusting for covariates. The risk of death among young children was higher in post-neonatal and children aged 1 to 4 years than in the neonatal age group, which was generally consistent with previous population-based studies in India and Nigeria [20, 23, 26]. The risk of under-five mortality in each separate age group was higher in households without a separate kitchen for cooking their meals compared to those with separate/outdoor kitchen. In addition, the lower risk of mortality was evident in children whose mothers ever breastfed compared to never breastfed children, consistent with its previously reported protective role in respiratory outcomes in young children [32, 34–36]. Unprocessed solid fuels when burnt in open fire contain a large amount of key pollutants such as fine particles, carbon monoxide (CO) and a number of other chemicals compared to clean (LPG/natural gas, biogas) cooking fuels, thus increases the risk of respiratory infections and death of children under-five years of age as air intake of an infant is approximately twice that of adults which result in inhaling more pollutants present into the indoor air [43].

This study is the first national evaluation of HAP and under-five mortality in Pakistan. The study found urban children were at slightly higher risk of death than children from rural areas, which was similar to previous similar studies in Bangladesh and Indonesia [24, 25]; despite the fact that the majority of the households in rural Pakistan rely on solids fuels mainly due to the unavailability of better alternatives [44, 45]. An intervention study in Pakistan investigating indoor particulate matter (PM) concentrations in developing countries reported that PM was considerably higher in urban kitchens as rural kitchens were more ventilated than urban ones [46]. Other studies from Pakistan have also reported that females were more likely to be responsible for cooking and that maternal education level and household wealth could influence the choice of household fuel type, with the usage of clean fuel lower in poorer households in both urban and rural areas [44, 45]. Similar findings were also evident in the present study for mortality outcomes associated with HAP.

Breastfeeding has previously been acknowledged to protect infants against infection and has been reported as a protecting factor for reducing the risk of respiratory illness among infants [32, 35, 36]. Thus breastfeeding status of children was investigated to determine whether it reduced the association between HAP and under-five mortality, a mechanism not previously investigated in any study in Pakistan. This present study identified that the risk of under-five mortality associated with the use of cooking fuel was higher in children aged 1 to 4 years compared to post-neonatal and neonatal age groups, and was stronger in children whose mother did not breastfeed. Breastfeeding in the first one year of life period may substantially reduce the risk of mortality among young children even among those exposed to HAP [23, 25, 26]. The recent PDHS data used in this study indicated that 94% of children were reported to have been breastfed at some time [1], that might be the cause of lower risk of neonatal and post-neonatal mortality associated with HAP in Pakistan.

A number of previous studies from developing countries (such as African countries, India, and Bangladesh) also examined the role of kitchen location in the house for the association between HAP and under-five mortality.[25, 26, 28] Findings from these studies were consistent with observed associations in the present study. Findings from the present study showed a greater risk of neonatal, post-neonatal, child and under-five mortality when mothers reported no separate kitchen in the house and used polluting fuels for cooking, and also found increased risk of childhood mortality even where clean fuels were used but no separate kitchen in the house. Households without a separate kitchen have a higher level of concentrations of PM, and young children are exposed to HAP as they spend many hours inside the house [47].

A number of methodological limitations that were taken into account when interpreting the results of this present study. First, the classification of cooking fuel may be a source of misclassification bias as some households use a combination of different fuels. The DHS only collected information of primary fuel use, no information on secondary fuel use was available. In addition, this study also did not account for past exposure to cooking fuel or recent changes in cooking methods. A recent study of rural areas in Pakistan indicated that majority of households used both fuels even where clean fuels were available. For example, households used LPG, but reduced fuel expenses by also using solid fuels as they were cheaper and more widely available (e.g. dung and crop residues) [45].

Second, there may be a source of recall bias as information on birth and death of children was based on interviews with mothers. Analyses were restricted to those children born within a five-year period prior to the survey date in order to minimise the likelihood of recall bias, but maximise the number of cases of death for analysis. An index period of 1-year period would reduce recall bias further, but also reduce the number of cases available for analysis; an index period of >5 years would increase the number of available cases for analysis, but also increase the likelihood of recall bias associated with those cases. It is also difficult to clearly define temporal relationships between the exposure (use of cooking fuel) and outcome (mortality) when collected at the same point in time, as the PDHS is cross-sectional design. Moreover, all-cause mortality was considered for our analyses of the association between HAP from cooking fuel and under-five mortality. There are also other important factors (such as preterm birth complications, low birth weight, nutritional conditions, and diarrhoea) along with respiratory illnesses that affect mortality among under-five children. However, cause-specific mortality could not be investigated in the present study due to the absence of this information in the PDHS dataset. Lower respiratory infection was the leading cause of death in children under five years of age in Pakistan [13] and HAP is mostly associated with respiratory illness. Nevertheless, counting all-cause mortality will also include mortality outcomes not associated with HAP from cooking fuel, which is likely to be a reason for ascertainment bias in the mortality outcomes which leads to an underestimation of the association between use of cooking fuel and the cause-specific outcomes noted above. In addition, this study also did not measure actual levels and patterns of exposure to emission from cooking smoke due to the lack of such objective measures in DHS data. Other important covariates, for example, “cooking under chimney” and “presence of windows in cooking area”, have been noted in previous studies [27, 28]; however, this information was not measured in the PDHS 2013 dataset.

Notwithstanding these methodological limitations, this present study was based on nationally representative survey data which has a very high response rate of 93.1%. This was also the first national level study in Pakistan to examine the association between HAP form cooking fuel and under-five mortality and assess the role of environmental and behavioural factors that may be points of effective intervention. The majority of the households in Pakistan still depend on polluting fuels (such as solid fuels, kerosene etc.) in both urban and rural areas [1, 2]; therefore, HAP remains a common exposure in the population and consequently, the population attributable risk of this avoidable risk factor remains an important public health issue for Pakistan.

Awareness-raising programs regarding the health risk associated with HAP from the use of polluting fuels need to be focused on rural and low-income urban areas of Pakistan. Changes in energy technologies such as switching to cleaner fuels (for example, LPG/natural gas, biogas, and electricity) are the key focus of interventions relating to HAP and can lead to a substantial reduction in exposure to a range of indoor air pollutants. However, cleaner fuel is not an affordable option for many poor families in Pakistan, and policies to increase access and use to cleaner fuels will require long-term intervention, investment in infrastructure and economic development of the country [3, 45, 46]. A shorter term and cost effective alternative to this problem is the use of improved/smoke-free cooking stoves (made of clay and husk), with a stack attached to the back of the stove to release smoke outside the kitchen, and which has been trialled in intervention studies in Pakistan [44–46, 48]. These cooking stoves are a simple and cheap intervention, and also have health advantages leading to a reduction in carbon monoxide emissions and incidence of respiratory infections [3, 44, 48].

Results from this present study suggest that behavioural and health educational interventions may potentially play a significant role in reducing childhood deaths in Pakistan associated with HAP. Such behavioural interventions include promoting exclusive breastfeeding practices in the first year of life and promoting the need to keep young children separate from the cooking area while cooking with solid fuels.

## Conclusions

This current study indicates that the ubiquitous use of polluting fuels in Pakistan is associated with an increased risk of mortality in children less than five years of age. Findings suggested that breastfeeding practices, cooking in a separate kitchen and not to carrying children while cooking may be potential targets for behavioural interventions and policy responses in Pakistan.

## Supporting information

S1 Table Distribution of study factors associated with under-five mortality in Pakistan, 2013 Pakistan Demographic and Health Survey (PDHS). (DOCX)
