# Supplementary material for: Household air pollution from use of cooking fuel and under-five mortality: The role of breastfeeding status and kitchen location in Pakistan
Source: PLoS One. 2017 Mar 9;12(3):e0173256. doi: 10.1371/journal.pone.0173256 (PMC5344381; doi:10.1371/journal.pone.0173256)
Supplement: S1 Table — (DOCX) [file pone.0173256.s001.docx]

**S1 Table. Distribution of study factors associated with under-five mortality in Pakistan, 2013 Pakistan Demographic and Health Survey (PDHS).**

| **Study factors** | **N** | **%** |
| --- | --- | --- |
| **Type of cooking fuel ^*^** |  |  |
| Electricity | 81 | 0.16 |
| LPG | 1839 | 3.73 |
| Natural Gas | 16725 | 33.93 |
| Biogas | 166 | 0.34 |
| Kerosene | 11 | 0.02 |
| Coal, lignite | 131 | 0.27 |
| Charcoal | 976 | 1.98 |
| Wood | 23,321 | 47.31 |
| Straw/shrubs/grass | 2079 | 4.22 |
| Animal dung | 3132 | 6.35 |
| **Place of residence** |  |  |
| Urban | 22131 | 44.89 |
| Rural | 27168 | 55.11 |
| **Wealth Index** |  |  |
| High income | 18,830 | 38.2 |
| Middle income | 9,867 | 20.01 |
| Low income | 20,602 | 41.79 |
| **Mother's age (years)** |  |  |
| <30 | 9762 | 19.80 |
| 30-39 | 19,763 | 40.09 |
| 40-49 | 19774 | 40.11 |
| **Mother's education** |  |  |
| No education | 33113 | 67.17 |
| Primary | 6136 | 12.45 |
| Secondary/higher | 10050 | 20.39 |
| **Mother's working status ^*^** |  |  |
| Working | 10858 | 22.02 |
| Not working | 38306 | 77.70 |
| **Sex of child** |  |  |
| Female | 23814 | 48.31 |
| Male | 25485 | 51.69 |
| **Breastfeeding Status** |  |  |
| Ever breastfed | 12587 | 25.53 |
| never breastfed | 36712 | 74.47 |
| **Household's floor material ^*^** |  |  |
| Cement/carpet | 26170 | 53.08 |
| Earth/Sand | 22280 | 45.19 |
| **Household's wall material ^*^** |  |  |
| Cement/brick | 30431 | 61.73 |
| Non-cement/non-brick | 18021 | 36.55 |
| **Separate kitchen** |  |  |
| Yes | 25945 | 60.57 |
| No | 16889 | 39.43 |
| **Smoking status of mother ^*^** |  |  |
| Yes | 3577 | 7.26 |
| No | 45668 | 92.74 |

^*^ Percentage did not add up to 100% because of missing values.
